# Foreign Body Ingestion in Neurologically Impaired Children: A Challenging Diagnosis and Management in Pediatric Surgery

**DOI:** 10.3390/children8110956

**Published:** 2021-10-23

**Authors:** Francesca Destro, Anna Maria Caruso, Cecilia Mantegazza, Luciano Maestri, Milena Meroni, Federica Pederiva, Mario Milazzo, Carlo Acierno, Gianvincenzo Zuccotti, Valeria Calcaterra, Gloria Pelizzo

**Affiliations:** 1 Pediatric Surgery Department, Children’s Hospital “Vittore Buzzi”, 20154 Milan, Italy; francesca.destro@asst-fbf-sacco.it (F.D.); luciano.maestri@asst-fbf-sacco.it (L.M.); milena.meroni@asst-fbf-sacco.it (M.M.); federica.pederiva@asst-fbf-sacco.it (F.P.); 2 Pediatric Surgery Unit, G. Di Cristina Children’s Hospital, 90121 Palermo, Italy; annacaruso81@gmail.com (A.M.C.); mario.milazzo@arnascivico.it (M.M.); carlo.acierno@arnascivico.it (C.A.); 3 Pediatric Department, Children’s Hospital “Vittore Buzzi”, 20154 Milan, Italy; cecilia.mantegazza@asst-fbf-sacco.it (C.M.); gianvincenzo.zuccotti@unimi.it (G.Z.); valeria.calcaterra@unipv.it (V.C.); 4 Department of Biomedical and Clinical Science “L. Sacco”, University of Milano, 20157 Milano, Italy; 5 Pediatrics and Adolescentology Unit, Department of Internal Medicine, University of Pavia, 27100 Pavia, Italy

**Keywords:** foreign body ingestion, children, disability, neurological impairment, pediatric surgery

## Abstract

Children with intellectual disability/neurodevelopmental delay (ID-ND) commonly ingest foreign bodies (FB) and often present complications due to peculiar aspects of their condition. The aim of this paper is to report the experience of two centers in the management of ID-ND patients after FB ingestion and to discuss a possible algorithm for clinical practice. We retrospectively evaluated data of patients managed for FB ingestion (period: 2017–2021), focusing on those with ID-ND, specifically demographics and baseline diagnosis, elements related to the event, symptoms, time to endoscopy, FB location, endoscopic details, and follow-up. A total of 457 patients were managed in the study period and 19 had ID-ND (mean age 9.8 ± 3.5 years, 15 males). A total of 16/19 (84.2%) were symptomatic and required an operative approach. Recurrent ingestions and multiple FB were found in 2 and 11 patients, respectively. Endoscopy (mean time 65.6 ± 41 min) was effective in 14 cases (73.6%) and 6 patients (31.6%) developed a complication. FB ingestion in ID-ND patients represents a challenging condition for the clinician and a potentially dangerous situation. It should be addressed specifically by a multidisciplinary team considering a tailored diagnostic and management protocol.

## 1. Introduction

Foreign body (FB) ingestion, including food bolus impaction, is a common pediatric issue that may occasionally require immediate intervention to avoid serious complications [1]. Data from the literature report an increasing annual rate of 91.5% from 1995 to 2015, with 18 per 10,000 children affected in 2015 [2]. The most common ingested objects are coins in more than 60% of children, followed by toys 10% and jewelry 7% [2].

FB ingestion occurs commonly accidentally in children aged between 3 months and 6 years [1]; at advanced ages, it may instead result from psychiatric disorders and intellectual disability/neurodevelopmental delay (ID-ND) [3,4]. Children belonging to the last group may not provide a correct clinical history, and if the event is unwitnessed, FB ingestion can be difficult to detect and may remain undiagnosed for a long time [4].

Fortunately, 80–90% of ingested FBs are naturally discharged within 4–6 days without untoward effects, while 10–20% may need to be removed endoscopically [1]. Nevertheless, some of them may lead to serious complications in the gastrointestinal system requiring surgical intervention [1]. Even seemingly harmless objects can cause serious and sometimes fatal injuries to internal organs, causing death [5].

Data from the National Institute of Statistics (ISTAT) showed that in 2010, 27% of the so-called “accidental” deaths in children aged 0–4 years were due to suffocation caused by ingestion/inhalation of a foreign body [5]. In children with ID-ND unexplained deaths by asphyxia, when not associated with epilepsy, occurred in those patients with a history of habitual feeding difficulties, bulimia, and cerebral palsy associated with severe spasticity. In a post mortem analysis, children with these problems were found to have airway obstruction caused by undigested food [6].

Long delays between ingestion to presentation and intervention might account for higher rates of surgery, perforation, and mortality in the group of patients with ID-ND [7]. Furthermore, besides the considerable patient morbidity, FB ingestion affects medical care resources and determines specific management challenges.

Herein we report challenging diagnosis/management occurring in children with ID-ND after ingestion of different FBs, at two pediatric surgery units. We discussed a possible algorithm for patient evaluation that could be proposed in clinical practice for the management of these patients.

## 2. Materials and Methods

Data were retrospectively analyzed from June 2017 to January 2021 of pediatric patients (≤18 years) referred to the Children’s Hospital V. Buzzi, Milano, Italy and Di Cristina Benfratelli Hospital, Palermo, Italy for FBI.

All data were collected from medical records of patients and included demographics, baseline diagnosis, living place, elements related to the event (witnessed or unwitnessed ingestion, type of FB), symptoms, the time between symptom onset and endoscopy, FB location, EGDS (esophago-gastro-duodenoscopy) duration and outcome. The need for surgery was also recorded.

The complication during follow-up was defined according to the modified Clavien Dindo classification [8].

### 2.1. Management Protocol

The management of patients was based on the clinical guidelines reported by the NASPGHAN (North American Society for Pediatric Gastroenterology, Hepatology and Nutrition) Endoscopic Committee in 2015 and ESPGHAN (European Society for Pediatric Gastroenterology, Hepatology, and Nutrition) in 2017 [9,10]. Endoscopic removal is required for esophageal FB and/or symptomatic patients. More aggressive treatment is indicated for multiple magnets (or a single magnet and a metallic object). Surgery is suggested in the case of complications such as bleeding, perforation, or fistula (esophageal button battery requires the involvement of representatives from cardiothoracic surgery and interventional cardiology). Sharp objects beyond the ligament of Treitz can be considered for surgical removal upon evaluation of benefits and risks.

### 2.2. Statistical Analysis

Qualitative variables were described as counts and percentages. Quantitative variables were expressed as the mean value when normally distributed. The association of categorical variables was assessed with chi-square or the Fisher’s exact test. A *p*-value below 0.05 was considered statistically significant.

The data analysis was performed with the STATA statistical package (release 15.1, 2017, Stata Corporation, College Station, TX, USA).

## 3. Results

### 3.1. Clinical Data, Signs, and Symptoms

A total of 457 (229 males, M, and 209 females, F) children were managed for FB ingestion. A total of 19 (15M/4F) out of 457 (4.2%) had ID-ND and autism (16 patients) or a syndrome (3 patients). Mean age at ingestion in children without ID-ND was lower compared to ID-ND patients (4.9 ± 3.9 vs. 9.8 ± 3.5 years, *p* < 0.01). A male predominance was noted in the ID-ND group (*p* = 0.02).

Considering patients with ID-ND, 16/19 (84.2%) were staying with their families at the time of ingestion, and 3 (15.8%) dwelled in a health care facility.

Sixteen patients (16/19, 84.2%) were symptomatic and complained of vomit (7/19, 36.8%), sialorrhea (5/19, 26.3%), thoracic or abdominal pain (4/19, 21%), gastrointestinal bleeding (2/19, 10.5%), intestinal obstruction (1/19, 5.3%), and persistent hiccup (1/19, 5.3%). One of them presented at the Emergency Department with a cardio-respiratory arrest that occurred abruptly in previously asymptomatic children and without evidence of FB ingestion.

The ingestion was witnessed by parents or caregivers in 8/19 patients (42%) but two of them eventually turned out to have also had unwitnessed events since we found multiple foreign bodies at endoscopy. In one case, the foreign body was accidentally found in the stomach during an endoscopic evaluation for celiac disease. Three asymptomatic patients (3/19, 15.7%, all males, mean age 11.6 ± 0.57 years) were managed conservatively after CT (computed tomography) evaluation that showed translucent FB in the ileum.

Two patients (1 male and 1 female, mean age 12.5 ± 0.7 years) had two episodes of ingestion in the same year, both requiring hospitalizations.

### 3.2. Operative Management

The stomach was the commonest site of FB location (8/19, 42.1%), followed by the esophagus (2/19, 1.0%), the duodenum (2/19, 1.0%), and the ileum (2/19, 1.0%). Four patients ingested multiple FBs that were found in different sites.

A total of 16 patients (84.2%, 4F/12M, mean age 9.8 ± 3.5 years) underwent endoscopy for FB removal. The details of the patients submitted to endoscopy are reported in Table 1. Mean time between symptom onset and endoscopy was 20.8 ± 25.5 h. Mean endoscopic time was 65.6 ± 41 min; in two cases (12.5%) surgery was additionally required.

**Figure 1:**
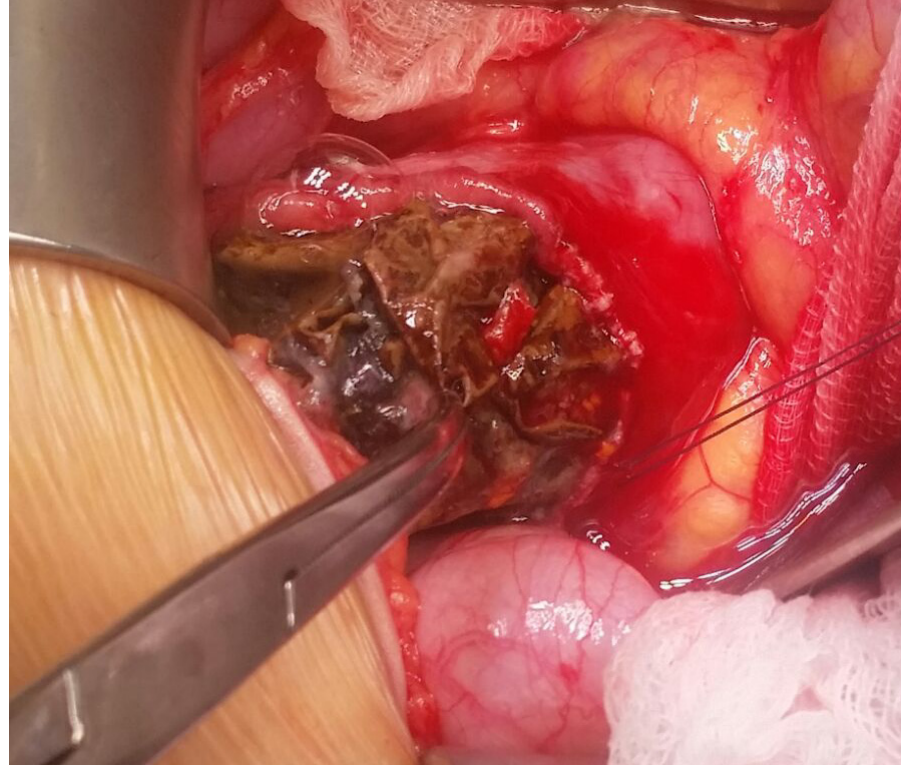
Intraoperative aspect of FB ingestion: removal of a scotch tape ball from the duodenum.

In particular, surgical removal of the FB was required in a 15-year-old boy with duodenal bezoar that resulted from unwitnessed and repeated ingestions of scotch tape. The boy underwent endoscopic evaluation 34 h after the admission with evidence of multiple inflammatory prepyloric polyps and duodenal bezoar that could not be removed despite several attempts with endoscopic graspers (total endoscopic duration 120 min). Open duodenotomy was then performed, and a huge ball of scotch tape was removed (Figure 1). The post-operative course was uneventful and endoscopic surveillance was performed after 3- and 6-months showing patent duodenum with regular mucosa.

**Figure 2:**
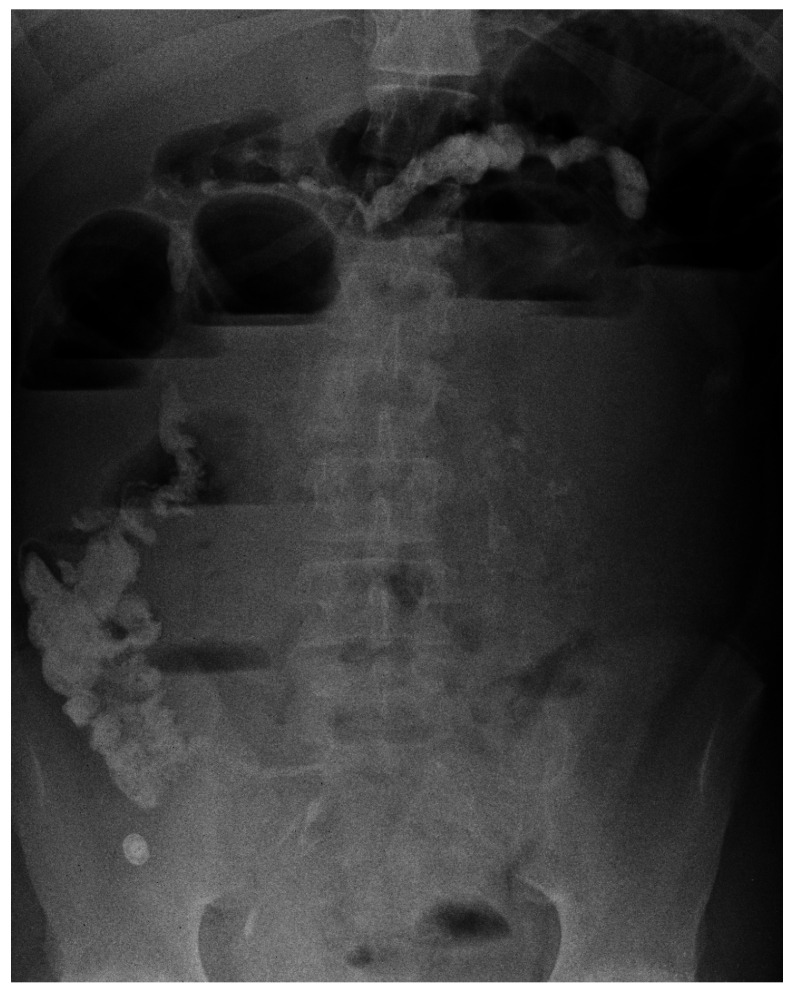
Abdominal X-ray and signs of intestinal obstruction.

The second patient was a 14-year-old adolescent affected by severe autistic syndrome with ND that presented acute abdominal pain and vomiting. He had a history of recurrent FB ingestions. At the last admission, laboratory tests were unremarkable, and imaging (ultrasounds, US), X-ray) was non-diagnostic. A few days later, the boy was readmitted because of intestinal obstruction with worsening of his clinical picture (Figure 2).

An abdominal CT scan, 12 h after admission, showed multiple FB in the gastric and ileal area. Therefore, he underwent EGDS with the removal of gastric and duodenal FB and residues (sponge, bezoar, plasticized foreign bodies, fishing line). After 72 h, he developed septic shock due to multiple intestinal perforations by FBs and died in the first post-operative day.

At follow-up, we had 6 complications (31.6%, 4M and 2F): 1 grade 5, 2 grade 2, 3 grade 1 according to modified Clavien Dindo classification.

## 4. Discussion

Children with ID-ND have the tendency to unintentionally ingest FB of various nature. Ingested FBs can be stuck anywhere in the gastrointestinal tract. Nature, size, and location of the FB affect the possibility of having symptoms and/or complications and the consequent management of patients.

According to Reilly et al., the higher risk of FB ingestion in ID-ND is related to several reasons, including poor control of hand-to-mouth activity and exploration of objects, prolonged oral phase, dysphagia with limited control over objects placed in the oral cavity, communication impairment, and altered protective mechanisms [11].

Our experience showed that 4.2% of ingestions occurred in ID-ND patients, an incidence higher than that reported by Khorana et al. [12]. We also found that ID-ND patients were significantly older compared to other children and, most commonly, males (78.9%). Otherwise, the literature data showed a similar involvement of both sexes (53.61% of males and 46.39% of females) [12]. Regarding age, it is known that the majority of accidental cases of FBI are seen in preschool age (in our series, mean age was 4.9 years, slightly more than the mean reported age of 3.6 years) [12], while intentional swallowing is primarily seen in adults with intellectual or mental disabilities, significant substance abuse, psychiatric disorders, or external motivations [13]. However, precise epidemiological data remain scarce.

Aberrant feeding behaviors have often been described in neurologically impaired children. As an example, food cravings were reported in ½ of autistic children compared to 2/10 of controls, with eating of nonfood substances affecting 33% of autistic children compared to only 3% of controls [6]. These data confirm that certain feeding habits or idiosyncrasies do indeed characterize developmentally disabled children. While these feeding habits seem to have little impact on the quality of the child’s diet, our case series demonstrates that they may have great consequences on the child’s safety [6].

As a matter of fact, the passage of the ingested FB through the gastrointestinal tract occurs in almost all pediatric cases without the need of external intervention, with endoscopy being required in 10–20% of cases and surgery in less than 1% [1]. On the other hand, our experience in patients with ID-ND showed spontaneous resolution in only 15.8% of the encountered cases with gastroscopy needed in all the other children and being effective in 87.5%. However, we cannot apparently know how many asymptomatic patients remained at their homes without requiring any evaluation; there may indeed be a number of ID-ND children who ingest FB and do not have complications or unwitnessed cases that remain unknown.

The diagnosis of FB ingestion is based on history and reported complaints (vomiting, abdominal pain, hematemesis, coughing, FB sensation, swallowing difficulties, sialorrhea, dyspnea).

In ID-ND patients, the ingestions often occur without any witnesses, the spectrum of the clinical picture is wide and non-specific, and there is a deficiency of reporting due to deficits in communication skills and affective behavior. Moreover, the nature of the FB can also deviate from the common ingested objects, and the event may be the effect of multiple ingestions. In our series, hairs, clothes, and sponges were the most common findings, followed by coins and pins; 11 patients (58%) ingested several pieces of different FB and 5 of them had agglomerates in the stomach, duodenum, and ileum. Attention should be paid to every object that can be potentially swallowed, especially those that are easily accessible, such as clothes.

Well-known and common comorbidities, such as sialorrhea and cough, represent elements that may further confuse the situation as well [14]. Therefore, FB may be identified only through an incidental radiograph for a seemingly unrelated chief complaint or accidentally visualized during endoscopy performed for other indications. Unfortunately, a delay in diagnosis may result in complications such as clinically evident respiratory distress (drooling, wheezing, or stridor) or even a cardio-respiratory arrest, as observed in one of our patients [15].

It is our duty to maintain a high degree of suspicion, to exclude possible biases such as those related to associated respiratory and gastrointestinal symptoms, and precisely evaluate the patient in order to deliver the most evidence-based diagnosis possible.

We recorded that mean time from symptom onset to endoscopy was more than 20 h. In a recent retrospective study, the maximum reported time from ingestion to the provision of medical attention was 2 weeks (range 5 min–2 weeks) but the authors did not extrapolate data of ID-ND patients, and no information on diagnostic delay from symptom onset to diagnosis is reported [11]. We speculate that this time becomes longer in ID-ND children due to unwitnessed ingestions.

Subtle hints are available for a clinician to help in the diagnosis of unwitnessed events, but they concern only unwitnessed esophageal FB [16] and are less relevant for children with ID-ND; age 2 years or younger, any physical examination finding of wheezes, retractions, rhonchi, or stridor, and fever 38.8 °C or higher have all been associated with an unwitnessed esophageal FB [17]. No hints for other locations can be found in the literature. Regardless, our patients were all older and did not show any of these symptoms.

It is important to consider the possibility of a FB ingestion in ID-ND patients, even when the anamnesis is unremarkable, and there was not a direct witness to the event. If a diagnosis is delayed or failed, severe health risks or even life-threatening situations may develop and could result in professional liability [5,7,18]. Complaints of clinical malpractice, inquiries, and litigation in the case of diagnostic delays and misdiagnoses may potentially occur.

Diagnostic delay has been pointed out as a factor worsening complications in FB ingestion [7]. Early upper endoscopy is reported to be the key to prompt diagnosis and management, especially in the case of esophageal FB [18]. X-ray is the most useful diagnostic tool in radio-opaque FB. However, in selected cases, diagnostic images, such as contrast X-ray and CT, may be useful in the management of children as well [19].

Even though we did not do any comparison with other patients managed for FB ingestion, the time lag between ingestion, symptom onset and diagnosis could be responsible of complicating the endoscopic procedure. Further studies are mandatory to confirm the data. The presence of experienced operators and a close-knit, trained team probably helped us to successfully complete the procedures in more than 85% of cases without any adverse event. Asymptomatic patients with witnessed events should be extensively evaluated with X-ray and CT scan when the FB cannot be identified on plain radiograph films. We noticed that the majority of our patients had a superior obstruction while only two cases presented with an ileal FB. This result could suggest that prior to CT evaluation, the contrast medium given by mouth may be helpful in detecting translucent FB in the upper gastrointestinal tract.

Clinicians should keep in mind that the spectrum of possible clinical scenario is wide, ranging from mild deviations from the usual behavioral (sudden food refusal, weight loss, changes in food patterns, and gradual or sudden onset of “difficult” mealtime behavior) to life-threatening situations, as we experienced in two patients [9]. In our series, vomit was the main symptom upon arrival (75%), while abdominal pain was present in only 6.2%, probably because it is not easily reported by ID-ND patients. Based on these results, surgery may be proposed in symptomatic patients or in children presenting with dilated bowel loops on X-ray, although the dilatation is often mild. The presented case series demonstrates the diagnostic and management difficulties surrounding FB ingestion in ND children and the need to always maintain a high level of suspicion. A prompt surgical approach, preferably minimally-invasive (MIS), for the high risks of repeated ingestions, reduces waiting times, and improves the outcome. Our experience also shows how difficult but crucial multi-professional diagnostic procedures and therapeutic interventions may be in the treatment of patients with ID-ND with a complex neuropsychiatric and medical picture. The management of a patient with ND and FB ingestion requires a team approach that incorporates endoscopic expertise and medical, surgical, and eventually neuropsychiatric support to provide the best patients care. Collaborative strategies across different disciplines may help prevent any issue that may be costly and burdensome both for the healthcare system and for the patient [11,14,20].

## 5. Conclusions

Our cases confirm that FBI in pediatric patients with ID-ND is rarely documented at admission. Their symptomatology can range from non-specific to life-threatening, time from symptom onset to diagnosis is prolonged and time from ingestion to admission is probably even longer. Intervention is necessary in most cases, with endoscopy being almost always effective with experienced operators but time-consuming. The peculiar aspects of ID-ND patients should be addressed specifically, with a dedicated diagnostic and multidisciplinary therapeutic management. In these patients, surgery, particularly MIS, requires early consideration.

## Figures and Tables

**Table 1:** Details of patients submitted to endoscopy.

Pt	Age (Years)	Sex	Baseline Diagnosis	Symptoms	Witnessed Ingestion	Symptom Onset-Endoscopy (Hours)	Type of FB	FB Location	EGDS Duration (min)	Surgery	Outcome
1	6	M	Autism	Vomit, sialorrhea	yes (only for coin)	5	Coin, hair, plastic toys	Esophagus (coin); stomach (others, casually identified)	40	no	Good
2	7	M	Autism	Abdominal pain, vomit	no	8	Button batteries; pins	Stomach	35	no	Good
3	9	M	Autism	Thoracic pain, vomit, hiccup	yes	4	Multiple coins	Stomach	40	no	Good
4	13	F	N/D	Vomit, sialorrhea, rectorrhagia	yes (only for battery)	48	Button battery, sponge, hair, fibers	Stomach, ileum	50	no	Pancreatitis
5	11	F	Autism	Vomit, hematemesis	no	3	Pins, bracelets	Stomach	80	no	Gastric bleeding
6	10	M	Autism	Vomit, sialorrhea	yes	6	Magnets (together)	Stomach	40	no	Good
7	14	M	Autism	Vomit, sub-obstruction	no	72	Multiple (sponge, bezoar, plasticized foreign bodies, fishing line)	Ileum	10	yes	Exitus
8	12	M	Autism	Vomit	no	24	Sponge, bezoar	Stomach, duodenum	50	no	Intestinal sub-obstruction
9	10	F	Autism	Asymptomatic	no	n/d	Hair, fibers	Stomach	30	no	Good
10	6	M	Autism	Thoracic pain, vomit, cough	yes	6	Plastic toys	Stomach	30	no	Good
11	9	M	Autism	Vomit, sialorrhea	no	12	Bezoar	Stomach	100	no	Intestinal sub-obstruction
12	15	M	N/D	Vomit	no	34	Scotch tape	Duodenum	120	yes	Good
13	8	M	Otahara syndrome	Cardiorespiratory arrest	no	6	Food (pear)	Esophagus	100	no	Good
14	16	F	Autism	Vomit	no	78	Clothes	Stomach and duodenum	110	no	Good
15	5	M	Autism	Vomit	yes	4	Dart	Stomach	150	no	Good
16	7	M	Autism	Sialorrhea	no	3	Coin	Esophagus	30	no	Good

## Data Availability

Data reported in this study are available upon request from the corresponding author.
